# Stigmatisation of mortuary workers in India: insights from four hospitals in West Bengal

**DOI:** 10.1016/j.lansea.2025.100559

**Published:** 2025-03-20

**Authors:** Saswata Sen, Rina Das, Tapobrata Guha Roy, Somnath Das

**Affiliations:** a R G Kar Medical College, Kolkata, West Bengal, India; b Department of Forensic Medicine and Toxicology, R G Kar Medical College, Kolkata, West Bengal, India; c Department of Community Medicine, R G Kar Medical College, Kolkata, West Bengal, India; d Department of Forensic Medicine & Toxicology, Bankura Sammilani Medical College & Hospital, Bankura, West Bengal, India

Death-care in hospitals is an area that has largely been neglected in the study of occupational hazards, working conditions, mental health, and social stigma faced by the staff working in this sector.^1^ The people working in hospital mortuaries, though invisible to the public, are involved in a profession often considered unhealthy, unhygienic, and repulsive in our society.^2^

Several studies have been conducted on the occupational health hazards of death-care workers^3^, ^4^, ^5^; and their mental and psychological health.^6^^,^^7^ However, there is evidence that the existence of discrimination can have detrimental effects on the social and mental well-being of these death-care workers. It can impede the effective functioning of the healthcare workforce, of which they are an integral part.^8^

A multi-centric study was conducted in four tertiary care teaching hospitals in West Bengal, India which explored the demographics and social stigma experienced by death-care workers in hospitals to assess prejudices and biases faced by attendants working in hospital mortuaries. We included 30 participants (all male) working in the mortuaries of the Forensic Medicine and Toxicology and Anatomy departments. Individuals aged ≥ 18 years and having a minimum of one year of work experience in a mortuary were included.

We used a quantitative research design, employing a 14-item questionnaire with a Likert scale. The interviewer-administered questionnaire was validated by five senior experts through a content validity index. We explored the experiences of death care workers through interviews, and the 12-point Likert scale items enabled the quantification of stigma perceptions.

Observations based on the Likert scale questions indicate a low mean response score, with statistically significant results (p-values < 0.001), suggesting that most death-care workers experienced rare instances of social stigma or discrimination (Fig. 1). However, discussions with respondents revealed recurring themes of individuals maintaining physical distance and displaying fear or avoidance towards them, with some not inviting them to social events. Furthermore, over half of the participating attendants provided inaccurate information about their occupation when enrolling their children in school, and one-third refrained from disclosing their occupation to others, indicating a lack of openness about their profession.

**Fig. 1:**
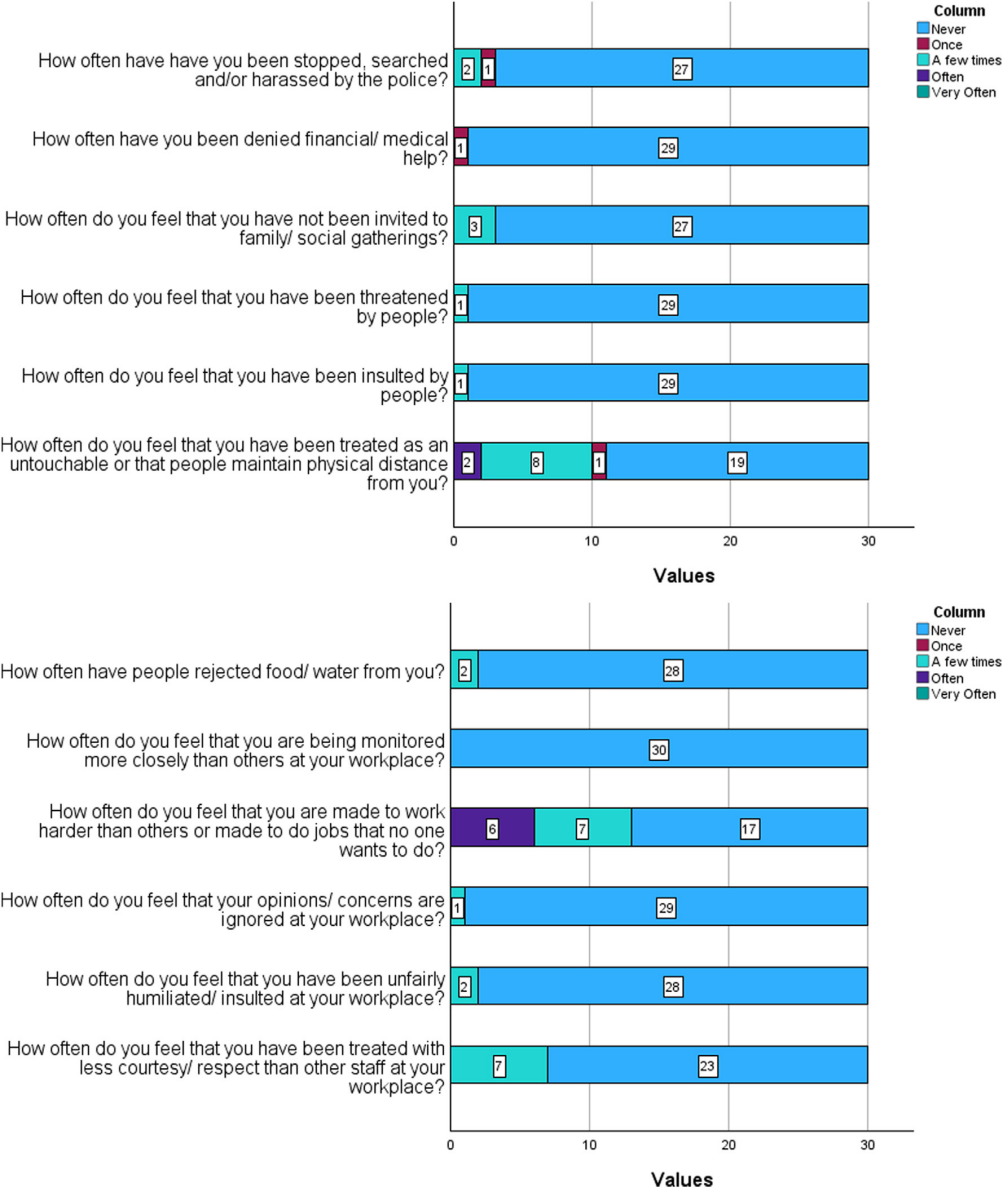
Graphical representation of responses to the Likert scale items.

Since death has always been a source of universal fear, people directly dealing with death suffer from social, professional, and psychological discrimination. This study has effectively underscored that a major part of the attendants working in hospital mortuaries have faced at least some form of discrimination (Supplementary Information). The implications of this study can be summarised as follows:

## Social discrimination

The fact that almost all participants are from Scheduled Caste and have some family members working in this field is enough proof of discrimination. This highlights the enduring nature of caste-based occupational segregation. Similarly, assurance of jobs within the community reduces the potential for educational upliftment. It is clear from the study that they have found it challenging to form or maintain social relationships beyond their professional environment.

## Professional challenges

There has been a lack of recognition and respect for the workers, who reported limited employment benefits. The proof that this occupation has passed through generations indicates limited job opportunities for the children of these death care workers. Stigmatisation also contributes to low wages and a poor working environment.

## Psychological impact

The internalisation of negative perceptions held by society has been a source of fear among these workers as they suppress their occupation from the community.

The nation should devise policies to minimise such marginalisation at the hospital and community levels. At the hospital level, re-naming of these professionals as “essential health workers”, improved workplace policies, and mental health support for the death care workers can be taken up. At the community level, creating a support system and professional associations by involving nonprofit organisations can be helpful. Reducing misconceptions, dispelling myths about the mortuaries and open discussion about the accurate and respectful role of the death care workers through media presentation can achieve the desired goal.

The literature reviewed in other parts of the world indicates that death-care workers who were seen as ‘less than human’ suffer psychological damage, cultural marginalisation, and self-stigma in the form of loss of friends or even spouses.^9^^,^^10^ Yet in times of crisis, such as the COVID-19 pandemic, they become almost indispensable for the entire nation in terms of death-care.^11^

To overcome the limitations of Likert scale-based studies, particularly in capturing the nuances of sensitive topics like stigma, future research can adopt a convergent mixed-methods design. This approach would balance depth and breadth, providing a comprehensive understanding of stigma among mortuary workers. By incorporating in-depth interviews alongside more objective measurement tools, researchers can gather richer insights into social stigma against death care workers, ensuring a more holistic and accurate portrayal of this complex issue.

## Contributors

SS: investigation, visualisation, writing—original draft, and software; RD: writing—review & editing, supervision, formal analysis, and data curation; TGR: data curation, formal analysis, methodology, validation, and software; SD: conceptualisation, methodology, data curation, formal analysis, supervision, writing—review & editing, and methodology.

## Declaration of interests

We declare no competing interests.

## References

[1] Guidetti G., Grandi A., Converso D. (2021). Funeral and mortuary operators: the role of stigma, incivility, work meaningfulness and work-family relation to explain occupational burnout. Int J Environ Res Public Health.

[2] Pinheiro F., Fischer F.M., Cobianchi C.J. (2012). Work of gravediggers and health. Work Read Mass.

[3] Beck-Sagué C.M., Jarvis W.R., Fruehling J.A., Ott C.E., Higgins M.T., Bates F.L. (1991). Universal precautions and mortuary practitioners: influence on practices and risk of occupationally acquired infection. J Occup Med Off Publ Ind Med Assoc.

[4] Akortiakumah J.K., Dartey A.F., Kuug A.K., Lotse C.W., Gnagmache G.K., Raji A.S. (2022). A qualitative exploratory study on the effects of formalin on mortuary attendants. SAGE Open Med.

[5] Suruda A., Schulte P., Boeniger M. (1993). Cytogenetic effects of formaldehyde exposure in students of mortuary science. Cancer Epidemiol Biomarkers Prev.

[6] Cotrim T., Soares G., Ferreira P., Barnabé R., Teles J., Prata N. (2020). Measuring psychosocial factors and predicting work ability among cemetery workers. Work Read Mass.

[7] Thorson J.A., Powell F.C. (1996 Jun). Undertakers' death anxiety. Psychol Rep.

[8] Nyblade L., Stockton M.A., Giger K. (2019 Feb 15). Stigma in health facilities: why it matters and how we can change it. BMC Med.

[9] Patwary M.A., O'Hare W.T., Elahi M.K., Hassan M.M., Sarker M.H. (2010). Domes and the dead. An example of extreme fatalism among mortuary workers in Bangladesh. Kaleidoscope.

[10] Dartey A.F., Dzansi G., Nyande F.K. (2024). Perception of dieners regarding social acceptance and the right to work: a qualitative study. Sage Open.

[11] Clavandier G., Berthod M.-A., Charrier P., Julier-Costes M., Pagnamenta V. (2021). From one body to another: the handling of the deceased during the COVID-19 pandemic, a case study in France and Switzerland. Hum Remains Violence.

