# Supplementary material for: Stigmatisation of mortuary workers in India: insights from four hospitals in West Bengal
Source: Lancet Reg Health Southeast Asia. 2025 Mar 20;35:100559. doi: 10.1016/j.lansea.2025.100559 (PMC11982943; doi:10.1016/j.lansea.2025.100559)
Supplement: Supplementary material [file mmc1.docx]

**Supplementary material**

**Stigmatisation of mortuary workers in India: insights from four hospitals in West Bengal**

*Saswata Sen^a^, Rina Das^a^, Tapobrata Guha Roy^a^, SomnathDas^a,b^**

*The Lancet Regional Health—Southeast Asia*

**Introduction to the study**

This research endeavour seeks to explore the comprehensive demographics and social stigma faced by death-care workers in hospital mortuaries, a topic that has garnered little attention in existing literature. The study aims to evaluate and present findings on social stigma, prejudices, and biases mortuary attendant’s encounter. Conducted across four tertiary care teaching hospitals in West Bengal, India—R G Kar Medical College, N R S Medical College, Midnapore Medical College, and Bankura Sammilani Medical College—the research focuses on understanding the experiences of these workers. Ethical clearance was obtained from the R G Kar Medical College Ethics Committee.

**Methods**

The study employed design, utilising Likert scale items and other closed-ended questions. A 14-item questionnaire was developed by the researchers, with its content validity assessed by five experts from the different departments. The questionnaire was complemented by interviews to gather additional insights. The use of a Likert scale allowed for the quantification of perceptions of stigma, enabling the differentiation between various levels of stigma rather than imposing a binary choice. This systematic approach facilitated straightforward statistical analysis and interpretation. All data collected were kept strictly anonymous and confidential to ensure participant privacy.

**Socio-demographic findings**

The socio-demographic analysis revealed that 29 out of 30 death-care workers (all males) had family members who had previously worked in mortuaries. Some attendants began their involvement in mortuary work as early as 16 years, indicating that this occupation often passes from father to child across generations. Despite this tradition, many death-care workers expressed a desire for their children to pursue higher-paying and more hygienic occupations.

**Experiences of social stigma**

The results from the Likert scale questions showed a low mean response score, with statistically significant findings (p-values < 0.001), suggesting that most death-care workers reported rare instances of social stigma or discrimination. However, the accuracy of these responses may be compromised due to the sensitive nature of the data and participants' reluctance to participate fully. This reluctance could stem from concerns about jeopardizing their professional reputation or facing social disapproval within their workplace, potentially introducing a social desirability bias. To mitigate this, participant anonymity was maintained, and confidentiality measures were communicated clearly.

**Caste-based considerations**

Notably, 28 of the 30 participating mortuary attendants belonged to Scheduled Castes (SC), with one each from the Other Backward Classes (OBC) and General category. This predominance of SC individuals in the occupation suggests a possibility of caste-based discrimination. Differentiating occupation-based stigma from caste-based stigma would require larger samples with a more diverse representation of castes.

**Supplementary Table 1: Socio-economic, demographic and work-related characteristics of the participants**

| **Characteristic** | | **Count** | **%** |
| --- | --- | --- | --- |
| Gender | Male | 30 | 100.0 |
|  | Female | 0 | 0.0 |
| Age (years) | Below 21 | 1 | 3.3 |
|  | 21 – 30 | 5 | 16.7 |
|  | 31 – 40 | 11 | 36.7 |
|  | 41 – 50 | 8 | 26.7 |
|  | Above 50 | 5 | 16.7 |
| Caste | General category | 1 | 3.3 |
|  | Scheduled Caste (SC) | 28 | 93.3 |
|  | Scheduled Tribe (ST) | 0 | 0.0 |
|  | Other Backward Class (OBC) | 1 | 3.3 |
| Education level | Below Grade 10 | 15 | 50.0 |
|  | Completed Grade 10 | 6 | 20.0 |
|  | Completed Grade 12 | 6 | 20.0 |
|  | College Graduate | 3 | 10.0 |
|  | Postgraduate | 0 | 0.0 |
| Wanted to pursue higher education? | No | 6 | 20.0 |
|  | Yes | 24 | 80.0 |
| Years of experience working in a mortuary | Below 6 | 7 | 23.3 |
|  | 6 – 15 | 11 | 36.7 |
|  | 16 – 25 | 6 | 20.0 |
|  | 26 – 35 | 5 | 16.7 |
|  | Above 35 | 1 | 3.3 |
| Did any family member work in a mortuary before? | No | 1 | 3.3 |
|  | Yes | 29 | 96.7 |
| Current area of residence | Rural | 6 | 20.0 |
|  | Urban | 24 | 80.0 |
| Marital status | Unmarried | 6 | 20.0 |
|  | Married | 24 | 80.0 |
|  | Divorced or Widower | 0 | 0.0 |
| Had any trouble getting married because of your occupation? | No | 30 | 100.0 |
|  | Yes | 0 | 0.0 |
| History of alcohol use or addiction | Absent | 10 | 33.3 |
|  | Present | 20 | 66.7 |
| Monthly income or expenditure (INR) | 10000 and below | 6 | 20.0 |
|  | 10001 – 20000 | 15 | 50.0 |
|  | 20001 – 30000 | 9 | 30.0 |
|  | Above 30000 | 0 | 0.0 |

Note: 1 US$ = 83.24 INR (on May 23, 2024)

**Supplementary Table 2: Responses to closed questions**

| Questions | | Count | % |
| --- | --- | --- | --- |
| **Q1**: Do you ever refrain from stating the exact nature of your occupation to people? | Yes | 10 | 33.3 |
|  | No | 20 | 66.7 |
| **Q2**: Did you provide accurate information about your occupation while admitting your children to school? | No | 17 | 56.7 |
|  | Yes | 7 | 23.3 |
|  | N/A (do not have children) | 6 | 20.0 |

**Supplementary Table 3: Responses to the Likert scale items**

| Items | Mean ± SD | Inter-Quartile Range (IQR) |
| --- | --- | --- |
|  |  |  |
| **Q3:** How often do you feel that you have been treated as an untouchable or that people maintain physical distance from you? | 0.77 ± 1.073 | 2 |
| **Q4:** How often do you feel that you have been insulted by people? | 0.07 ± 0.365 | 0 |
| **Q5:** How often do you feel that you have been threatened by people? | 0.07 ± 0.365 | 0 |
| **Q6:** How often do you feel that you have not been invited to family or social gatherings? | 0.20 ± 0.610 | 0 |
| **Q7:** How often have you been denied financial or medical help? | 0.03 ± 0.183 | 0 |
| **Q8:** How often have you been stopped, searched and/or harassed by the police? | 0.17 ± 0.531 | 0 |
| **Q9:** How often do you feel that you have been treated with less courtesy or respect than other staff at your workplace? | 0.47 ± 0.860 | 0 |
| **Q10:** How often do you feel that you have been unfairly humiliated or insulted at your workplace? | 0.13 ± 0.507 | 0 |
| **Q11:** How often do you feel that your opinions or concerns are ignored at your workplace? | 0.07 ± 0.365 | 0 |
| **Q12:** How often do you feel that you are made to work harder than others or made to do jobs that no one wants to do? | 1.07 ± 1.285 | 2 |
| **Q13:** How often do you feel that you are being monitored more closely than others at your workplace? | 0.00 ± 0.000 | 0 |
| **Q14:** How often have people rejected food and/or water from you? | 0.13 ± 0.507 | 0 |

**Supplementary Table 4: Responses to follow-up questions**

| Follow up questions | | Count | % |
| --- | --- | --- | --- |
| If given a chance, would you like to change your occupation? | No | 11 | 36.7 |
|  | Yes | 19 | 63.3 |
| Would you want any of your children or family member to work in a mortuary in the future? | No | 20 | 66.7 |
|  | Yes | 10 | 33.3 |
